# Supplementary material for: Sleep Disturbances and Mental Well-Being of Preschool Children during the COVID-19 Pandemic in Mexico
Source: Int J Environ Res Public Health. 2023 Mar 1;20(5):4386. doi: 10.3390/ijerph20054386 (PMC10002191; doi:10.3390/ijerph20054386)
Supplement: Supplementary file 1 [file ijerph-20-04386-s001.zip › Supplementary file S1.pdf]

Preschool-Age Children Sleep Disturbances and Mental Well-Being During COVID-19 Pandemic.

The following questions relate to your children's dynamics, activities, sleep, and mental well-being during the COVID-19 pandemic. Please, fill up the form as accurately and completely as possible.

Section 1: Parent Information

- Full name:
  -

- What is your gender?
  - Female
  - Male
  - Other

- What is your kinship to the child participating in the study?
  - I am her/his mother
  - I am her/his father
  - I am her/his tutor

- What is your marital status?
  - Single
  - Married
  - Free union
  - Separated
  - Divorced

- What is your age?

- What is your school level?
  - Middle school
  - High school
  - Undergraduate
  - Master’s degree
  - PhD
  - Specialization

- What is your occupation?

- Do you suffer from any disease? Which one(s)?

- Do you have a sleep disorder?
  - Yes
  - No

- If you responded "yes" to the previous question, do you take any medication for it? Which one(s)?

- Have you been diagnosed with a mental health disorder (Example: Depression, anxiety, attention deficit hyperactivity disorder)?
  - No
  - Depression
  - Anxiety
  - Attention deficit hyperactivity disorder
  - Personality disorders
  - Other

- If you have been diagnosed with a mental health disorder, do you take any medication for it? Which one(s)?

Section 2: Children Information

Answer the following sections with the information of your daughter or son participating in the study:

- Gender
  - Male

  - Female
  - Other

- Bodyweight in kilograms

- Height in centimeters

- Date of birth

- Age in years and months

- Way of birth
  - C-section
  - Natural birth

- Was the child born prematurely?
  - Yes
  - No

- Is your daughter/son an only child?
  - Yes
  - No

- What number of daughter/son is she/he?
  - First
  - Second
  - Third
  - Fourth

    - Fifth
    - Other

- How many children live in the household?
    - 1
    - 2
    - 3
    - 4
    - 5
    - Other

- What grade of school is the child currently in?
    -

- How many days a week does the child take classes?
    - 1
    - 2
    - 3
    - 4
    - 5
    - 6
    - 7

- How many hours does the school day last?
    - 1
    - 2
    - 3
    - 4
    - 5
    - 6

- Does your child participate in any extracurricular activities?
  Yes
  No

- If the answer to the last question was "yes" how many hours a day were spent on the activity?

- What is your child's confinement status to this day?

- What modality of school attendance does your child is taking now?

  - Online platform
  - T.V. classes
  - Face-to-face attendance
  - Not currently on school
  - Other

- Does the child have from any disease?
  - Yes
  - No

- If you responded yes to the previous question, which one(s)?

- Does the child suffer from recurrent respiratory disease?
  - Yes
  - No
- Does the child have food or drug allergies?
  - Yes
  - No

- If you responded yes to the previous question, which allergies?

- Does the child have allergic rhinitis or seasonal allergies?
  - Yes
  - No

- Does the child have any mental health related diagnosis?

- He/she doesn't have any diagnosis
- Anxiety
- Autism
- Attention deficit hyperactivity disorder
- Other

- In the event of having a mental health diagnosis, how long has it been since the diagnosis?

- In the event of having a mental health diagnosis, does the child receive any treatment? Which?

- Does the child have a neurological disorder?
  - No diagnosis
  - Epilepsy
  - Other

- In the event of having a neurological diagnosis, does the child receive any treatment? Which?

- Has the child been hospitalized in the last 12 weeks?
  - Yes
  - No

- Has the child been sick in the last 12 weeks?
  - Yes
  - No

- Does the child currently take any medication of any kind? (Including over-the-counter vitamins and supplements)
    - o Yes
    - o No

- If you responded Yes to the previous question, write the medication(s) and the dose he or she takes.

Electronics Use

During the COVID-19 Pandemic, your children's electronic use (excluding scholarly use) has:

- Increased
- Decreased
- Remain the same

- Does the child have a television in his or her bedroom?
    - o Yes
    - o No

- Does the child have a tablet in his or her bedroom?
    - o Yes
    - o No

- Does the child watch television close to bedtime? (at least 2 hours before)
    - o Yes
    - o No

- Does the child use the tablet close to bedtime? (at least 2 hours before)
    - o Yes
    - o No

- How many hours a day does the child watch television?
    - 0 or less than an hour a day 1 2 3 4 5 6 7 8 9 10 or more

- How many hours a day does the child use the computer or laptop?
    - 0 or less than an hour a day 1 2 3 4 5 6 7 8 9 10 or more

- How many hours a day does the child use the Tablet or "iPad"?
    - 0 or less than an hour a day 1 2 3 4 5 6 7 8 9 10 or more

- How many hours a day does the child use a cell phone? Uses: watching videos, playing mobile games, viewing photos.
    - 0 or less than an hour a day 1 2 3 4 5 6 7 8 9 10 or more

- In an average week, how long does your child spend:

**Watching TV and movies:**	0 or less than an hour a week	Less than 5 hours a week	Between 5 and 10 hours a week	Between 10 and 15 hours a week	More than 15 hours a week
**Using a tablet:**	0 or less than an hour a week	Less than 5 hours a week	Between 5 and 10 hours a week	Between 10 and 15 hours a week	More than 15 hours a week
**Using computers:**	0 or less than an hour a week	Less than 5 hours a week	Between 5 and 10 hours a week	Between 10 and 15 hours a week	More than 15 hours a week
**Using a smartphone:**	0 or less than an hour a week	Less than 5 hours a week	Between 5 and 10 hours a week	Between 10 and 15 hours a week	More than 15 hours a week

Child's Sleep Habits (Preschool and School-Aged)

The following statements are about your child's sleep habits and possible difficulties with sleep. Think about the past week in your child's life when answering the questions. If last week was unusual for a specific reason (such as your child had an ear infection and did not sleep well or the TV set was broken), choose the most recent typical week. Answer USUALLY if something occurs 5 or more times in a week; answer SOMETIMES if it occurs 2-4 times in a week; answer RARELY if something occurs never or 1 time during a week. Also, please indicate whether or not the sleep habit is a problem by circling "Yes," "No," or "Not applicable (N/A).

Bedtime

Write in child´s bedtime:

	Usually (5-7)	Sometimes (2-4)	Rarely (0-1)	Problem?
1. Child goes to bed at the same time at night (R)				Yes No N/A
2. Child falls asleep within 20 minutes after going to bed (R)				Yes No N/A
3. Child falls asleep alone in own bed (R)				Yes No N/A
4. Child falls asleep in parent's or sibling's bed				Yes No N/A
5. Child needs parent in the room to fall asleep				Yes No N/A
6. Child struggles at bedtime (cries, refuses to stay in bed, etc.)				Yes No N/A
7. Child is afraid of sleeping in the dark				Yes No N/A
8. Child is afraid of sleep alone				Yes No N/A

Sleep Behavior

Child's usual amount of sleep each day: ____________ hours and ____________ minutes (combining nighttime sleep and naps)

	Usually (5-7)	Sometimes (2-4)	Rarely (0-1)	Problem?
9) Child sleeps too little				Yes No N/A

10) Child sleeps the right amount (R)				Yes No N/A
11) Child sleeps about the same amount each day (R)				Yes No N/A
12) Child wets the bed at night				Yes No N/A
13) Child talks during sleep				Yes No N/A
14) Child is restless and moves a lot during sleep				Yes No N/A
15) Child sleepwalks during the night				Yes No N/A
16) Child moves to someone else's bed during the night (parent, brother, sister, etc.)				Yes No N/A
17) Child grinds teeth during sleep (your dentist may have told you this)				Yes No N/A
18) Child snores loudly				Yes No N/A
19)Child seems to stop breathing during sleep				Yes No N/A
20) Child snorts and/or gasps during sleep				Yes No N/A
21) Child has trouble sleeping away from home (visiting relatives, vacation)				Yes No N/A
22) Child awakens during night screaming, sweating, and inconsolable				Yes No N/A
23) Child awakens alarmed by a frightening dream				Yes No N/A

Waking During the Night

	Usually (5-7)	Sometimes (2-4)	Rarely (0-1)	Problem?
24) Child awakes once during the night				Yes No N/A
25) Child awakes more than once during the night				Yes No N/A

Write the number of minutes a night waking usually lasts: ________________

Morning Waking/Daytime Sleepiness

Write in the time-of-day child usually wakes in the morning: ______________

	Usually (5-7)	Sometimes (2-4)	Rarely (0-1)	Problem?
26) Child wakes up by him/herself (R)				Yes No N/A
27) Child wakes up in negative mood				Yes No N/A
28) Adults or siblings wake up child				Yes No N/A
29) Child has difficulty getting out of bed in the morning				Yes No N/A
30) Child takes a long time to become alert in the morning				Yes No N/A
31) Child seems tired				Yes No N/A

Child has appeared very sleepy or fallen asleep during the following (check all that apply):

	Not Sleepy	Very Sleepy	Falls Asleep
32) Watching TV			
33) Riding in car			

Owens JA, Spirito A, McGuinn M. The children's sleep habits questionnaire (CSHQ): psychometric properties of a survey instrument for school-aged children. Sleep 2000, Dec 15;23(8):1043-51

Material directly provided by contacting the author.

Strengths and Difficulties Questionnaire (SDQ) double-sided version with impact supplement for parents of 2-4 years old and for parents of 4-17 years old is currently available free of charge at its official website: https://www.sdqinfo.org/
